# Projected health benefits of air pollution reductions in a Swedish population

**DOI:** 10.1177/14034948241264099

**Published:** 2024-11-26

**Authors:** Anna Oudin, Erin Flanagan, Bertil Forsberg

**Affiliations:** 1 Division of Sustainable Health, Department of Public Health and Clinical Medicine, Umeå University, Umeå, Sweden; 2 Division of Occupational and Environmental Medicine, Department of Laboratory Medicine, Lund University, Lund, Sweden

**Keywords:** Health impact assessment, ambient air pollution, PM_2.5_, NO_2_, clean air policy, mortality, morbidity

## Abstract

**Background:**

A large part of the Swedish population is exposed to higher levels of air pollution than the health-centered air quality guidelines recommended by the World Health Organization (WHO).

**Aim:**

The aim of the study was to illustrate the potential health benefits of cleaner air in Sweden by conducting a comprehensive health impact assessment, using a population sample of 100,000 individuals representing the country’s demographics.

**Methods:**

Exposure-response functions for various health outcomes were derived from epidemiological literature, mainly from systematic reviews and low-exposure settings. Two hypothetical scenarios were studied: a 1 µg/m^3^ decrease in particulate matter with an aerodynamic diameter <2.5µm (PM_2.5_) and nitrogen dioxide (NO_2_), and a reduction in PM_2.5_ or NO_2_ from average exposure corresponding to Sweden’s Clean Air objectives to WHO’s air quality guidelines.

**Results:**

The findings demonstrated that even a modest decrease in air pollution concentrations can yield significant health benefits. For example, reducing PM_2.5_ by 1 µg/m^3^ was projected to correspond to a 1% to 2% decrease in mortality, a 2% reduction in myocardial infarction cases, a 4% decrease in stroke incidence, a 2% decline in chronic obstructive pulmonary disease, and a 1% decreases in lung cancer and type 2 diabetes annually. Moreover, this reduction is estimated to lower childhood asthma cases, incidences of hypertension during pregnancy, and premature births by 3%, 3% and 2%, respectively, each year.

**Conclusions:**

**The results highlighted that even minor enhancements in air quality would lead to substantial improvements in public health.**

## Introduction

Exposure to ambient air pollution is the largest environmental public health burden leading to approximately 4.2 million deaths each year [1]. Sweden, located in Northern Europe, is one of the countries with the cleanest air in the world. Nonetheless, air pollution contributes to about 6700 premature deaths each year according to the most recent estimate [2]. Air pollutants, including both particulate matter (PM) with an aerodynamic diameter <2.5µm (PM_2.5_) and <10 (PM_10_) as well as nitrogen dioxide (NO_2_), have been associated with mortality and morbidity in Sweden, despite the country’s relatively low levels of air pollution. Consequently, air pollution remains a significant public health issue.

Recognizing the growing evidence of air pollution’s negative health impacts, the World Health Organization (WHO) revised their air quality guidelines in September 2021 [3], encompassing a drastic decrease from their earlier 2005 values [4] (Table I). Air pollution levels now exceed WHO’s air quality guidelines in more than 99% of world regions. These updated health-centered guidelines are also notably lower than Sweden’s own legally binding environmental quality standards (*Miljökvalitetsnormer*, MKN) [5] for PM_2.5_ and NO_2_ as well as Sweden’s more ambitious, yet non-binding, environmental quality objective on air quality, Clean Air (*Miljömål*, *Frisk Luft*) [6] (Table I). The MKN do not adequately prevent illness and safeguard public health, as existing evidence shows associations with adverse health even below these levels. The Clean Air objective declares that “the air shall be so clean so that human health as well as animals, plants and cultural values are not harmed” and claims its guidelines protect even the most vulnerable in society. Although progress has been made toward achieving the Clean Air objective, its conditions have not been met everywhere. With this, much more work is needed to ensure clean air for all.

**Table I.** Annual mean guideline value (µg/m^3^) for PM_2.5_ and NO_2_ according to the World Health Organization’s (WHO) air quality guidelines (2005 and 2021) as well as Sweden’s environmental quality standard (MKN) and environmental quality objective (Clean Air).

Air pollutant	WHO’s 2005 air quality guideline	WHO’s 2021 air quality guideline	Sweden’s MKN	Sweden’s Clean Air objective
PM_2.5_	10	5	25	10
NO_2_	40	10	40	20

There is thus a need for a clearer foundation upon which to communicate the potential health benefits of cleaner air in Sweden. The aim of the present study was, therefore, to compose such a communication tool in the form of a health impact assessment (HIA) that estimates and compares the health effects of hypothetical air quality reduction scenarios, including a decrease in air pollution concentrations by 1 µg/m^3^, and a decrease from Sweden’s Clean Air objective to the WHO’s 2021 air quality guidelines.

## Materials and methods

### Study setting

**Figure 1.**
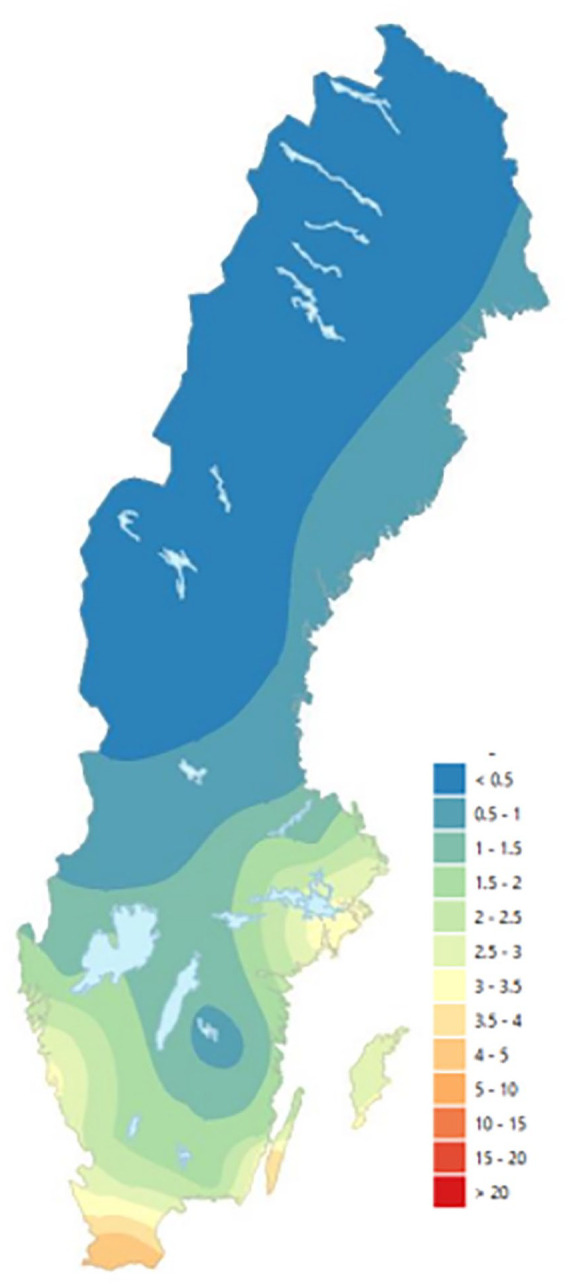
2019 annual mean regional background concentrations of PM_2.5_ in Sweden, unit µg/m^3^. Image derived from Gustafsson et al. [2].

Sweden, located in Northern Europe, is one of the countries with the cleanest air in the world. Nonetheless, it is estimated that air pollution contributes to about 6700 premature deaths each year [2]. Concentrations of PM_2.5_ are higher further south in Sweden (Figure 1) because these regions are affected by particulate matter from the rest of Europe to a larger degree. In southern Sweden, for instance, a large proportion of total PM_2.5_ concentrations consists of long-range, in-transported particles. The contribution of local sources to total PM_2.5_ concentrations, on the other hand, is generally quite low in Sweden overall.

### Study population

The study population was based on the demographic structure of 100,000 people as well as the average age distribution of Sweden’s entire population in 2019, derived from Statistics Sweden (Table S1, Supplemental materials). From this, the number of persons in different age groups and risk groups could be calculated.

### Health impact assessment

The following standard formula was used for the health impact analysis:

$$\Delta Y=Y_{0}.\left(1-e^{-\beta \Delta x}\right)$$

where *ΔY* is the change in health outcome, *Y_0_* is the baseline health incidence, current/baseline frequency, or prevalence of the health outcome, *ß* is the exposure-response function (log of the relative risk, assuming linear exposure-response functions), and *Δx* is the hypothetical change in air pollution exposure.

Analyses were conducted using SPSS Statistical Software version 25 (IBM^®^, Armonk, NY, USA).

#### Baseline health outcomes: incidence

The health outcomes included in the present study were all-cause mortality, myocardial infarction, stroke, chronic obstructive pulmonary disease (COPD), lung cancer, type 2 diabetes, childhood asthma, hypertensive disorders of pregnancy (HDP), and preterm birth. These were selected based on a review, conducted by the authors for the Swedish Environmental Protection Agency (EPA) in 2020, of the evidence for health outcomes associated with air pollution exposure, with a particular focus on evidence from low-level areas. This review was based on reports by the European Commission and WHO, “Health risks of air pollution in Europe” and “Review of evidence on health aspects of air pollution”; a joint statement from the American Thoracic Society and European Respiratory Society; and the United States (U.S.) EPA Integrated Science Assessments (ISA). Outcomes for which the evidence of the association was deemed to be a causal relationship or likely to be a causal relationship were selected. Details on the baseline health incidence, baseline frequency, or prevalence of each health outcome are presented in Table II.

**Table II.** Assumed base frequencies (for year 2019 unless otherwise stated) of each investigated health outcome, along with their corresponding age groupings, source, and additional information.

Health outcome	Incidence per 100,000	Source	Additional comments
All-cause mortality ⩾30 years of age	1329	Statistical database of SNBHW	Sweden’s population
Myocardial infarction ⩾30 years of age	275	Statistical database of SNBHW	First incident acute myocardial infarction after 7 years without myocardial infarction
Stroke ⩾30 years of age	303	Statistical database of SNBHW	First incident stroke after 7 stroke-free years
Chronic obstructive pulmonary disease (COPD) ⩾50 years of age	157	Lindberg et al., 2006 [7]	A regional study based in Norrbotten, Sweden, year 1996
Lung cancer ⩾35 years of age	72	Statistical database of SNBHW	Primary lung cancer including the bronchi, irrespective of tumor type
Type 2 diabetes ⩾15 years of age	400	Norhammar et al., 2016 [8]	Sweden’s population, year 2012
	Risk		
Childhood (2–18 years of age) asthma*	7.5% per childhood	Oudin et al., 2017 [9]	National study
Hypertensive disorders of pregnancy All pregnant women	2.8% per pregnancy	Khashan et al., 2019 [10] and statistical database of SNBHW	Prevalence from all singleton births in Sweden from 1982–2012; 116,071 births, year 2019
Preterm birth** All pregnant women	5.6% per pregnancy	Statistical database of SNBHW	Sweden’s population and 116,071 births, year 2019

*Note*. SNBHW = Swedish National Board of Health and Welfare (*Socialstyrelsen*).

* With prescribed medication. **Gestation ⩽36 weeks.

#### Exposure-response functions

Exposure-response functions (ERFs) were based on epidemiological literature; evidence from systematic reviews and meta-analyses were prioritized where possible. These are described in Table III.

**Table III.** Assumed exposure-response functions (ERFs) and their sources for each investigated health outcome by pollutant.

Health outcome	PM_2.5_	NO_2_
All-cause mortality ⩾30 years of age	A) 1.08 per 10 µg/m^3^ (Chen and Hoek [11])^1^ B) 1.26 per 10 µg/m^3^ (Turner et al., [12])^2^	1.05 per 10 ppb (Stieb et al., [13])^3^
Myocardial infarction ⩾30 years of age	1.13 per 5 µg/m^3^ (Cesaroni et al., [14])^4^	1.03 per 10 µg/m^3^ (Gandini et al., [15])^5^
Stroke ⩾30 years of age	1.10 per 5 µg/m^3^ (Wolf et al., [16])^6^	1.08 per 10 µg/m^3^ (Wolf et al., [16])^6^
Chronic obstructive pulmonary disease (COPD) ⩾50 years of age	1.18 per 10 µg/m^3^ (Park et al., [17])^7^	1.07 per 10 µg/m^3^ (Park et al., [17])^7^
Lung cancer ⩾35 years of age	1.11 per 10 µg/m^3^ (Ciabattini et al., [18])^8^	1.04 per 10 µg/m^3^ (Hamra et al., [19])^9^
Type 2 diabetes ⩾15 years of age	1.25 per 10 µg/m^3^ (He et al., [20])^10^	
Childhood asthma* 2–18 years of age	1.03 per 1 µg/m^3^ (Khreis et al., [21])^11^	1.05 per 4 µg/m^3^ (Khreis et al., [21])^11^
Hypertensive disorders of pregnancy All pregnant women	1.32 per 10 µg/m^3^ (Yu et al., [22])^12^	
Preterm birth** All pregnant women	1.24 per 10 µg/m^3^ (Klepac et al., [23])^13^	1.09 per 10 µg/m^3^ (Klepac et al., [23])^13^

*Note*. ppb: parts per billion.

1 Review of 104 cohort studies; ^2^American cohort study; ^3^review of 47 cohort studies; ^4^review of 11 cohorts from the European Study of Cohorts for Air Pollution Effects (ESCAPE) project; ^5^national Italian study; ^6^review of 12 cohort studies for PM_2.5_ (concentrations <15 µg/m^3^) and 12 cohort studies for NO_2_; ^7^review of 7 cohort studies; ^8^review of 4 cohort studies for PM_2.5_ and lung cancer incidence as well as 7 cohorts studies for PM_10_ (both lung cancer incidence and mortality); ^9^review of 20 studies (both lung cancer incidence and mortality); ^10^review of 8 cohort studies, ^11^ review of 41 cohort studies; ^12^review of 9 studies; ^13^review of 48 studies (both cross-sectional and longitudinal).

* With prescribed medication. **Gestation of ⩽36 weeks.

A blank cell denotes that insufficient evidence existed within the literature to confidently derive an ERF.

For all-cause mortality, two different ERFs were utilized because evidence suggests that the strength of an association varies depending on how detailed the air pollutant model and/or its proximity to the source [24]. To address this uncertainty, both the standard ERF (1.08 per 10 µg/m^3^) derived from a 2020 systematic review and meta-analysis used by the WHO in their 2021 air quality guideline revision [3, 11] and an ERF that better describes the relationship between near-source (or locally produced) PM_2.5_ and mortality (1.26 per 10 µg/m^3^) were employed [12].

#### Hypothetical change in air pollution exposure

Two hypothetical air pollution reduction scenarios were investigated. In Scenario 1, the 1 µg/m3 reduction was applied to the population’s actual exposure to PM_2.5_ and NO_2_. Scenario 2 assumed that the entire study population was exposed to average air pollution concentrations corresponding to that of Sweden’s Clean Air objective (10 and 20 µg/m3 for PM_2.5_ and NO_2_, respectively) at baseline, which was then reduced to an average exposure corresponding to the WHO’s 2021 air quality guidelines (5 µg/m^3^ for PM_2.5_ and 10 µg/m^3^ for NO_2_). PM_10_ was not included in this investigation, as there is less evidence on its long-term health impacts compared with PM_2.5_ and NO_2_.

## Results

Estimated health impacts for the two hypothetical air pollution reduction scenarios are shown in Table IV.

**Table IV.** Estimated health impacts, described as the number of prevented cases (*N*) per year, within each relevant age group for a population of 100,000 people with the same age distribution as Sweden’s total population following each of the two hypothetical air pollution reduction scenarios.

	Scenario 1 (1 µg/m^3^ reduction) *N* (%)	Scenario 2 (Swedish Clean Air objective to WHO 2021 guidelines^1^) *N* (%)
PM_2.5_		
All-cause mortality		
A) 1.08 per 10 µg/m^3^ (Chen and Hoek, 2020) [25]	10 (1)	50 (4)
B) 1.26 per 10 µg/m^3^ (Turner et al., 2016) [12]	30 (2)	
Myocardial infarction	7 (2)	32 (12)
Stroke	11 (4)	51(17)
COPD	3 (2)	13 (8)
Lung cancer	0.8 (1)	4 (5)
Type 2 diabetes	4 (1)	19 (5)
Childhood asthma*	14 (3)	64 (14)
HDP	0.9 (3)	4 (13)
Preterm birth**	1 (2)	6 (10)
NO_2_		
All-cause mortality	10 (1)	99 (7)
Myocardial infarction	3 (1)	8 (3)
Stroke	2 (1)	22 (7)
COPD	1 (1)	10 (7)
Lung cancer	0.3 (0.4)	3 (4)
Childhood asthma*	6 (1)	54 (11)

COPD: chronic obstructive pulmonary disease; HDP: hypertensive disorders of pregnancy.

* 2–18 years of age with prescribed medicine. **Gestation of ⩽36 weeks.

1 Scenario 2 corresponds to a 5 µg/m^3^ reduction in PM_2.5_ and a 10 µg/m^3^ reduction in NO_2_ concentrations.

The results illustrated that even a small reduction in air pollution concentrations had the potential to yield significant health gains. For instance, a 1 µg/m^3^ reduction (Scenario 1) of PM_2.5_ was estimated to lead to a 1% to 2% decrease in mortality, a 2% decrease in cases of myocardial infarction, a 4% decrease in stroke incidence, a 2% decrease in COPD, and a 1% decrease in both lung cancer and type 2 diabetes, annually. Additionally, the number of children with asthma, cases of HDP, and premature birth incidences were estimated to decrease by 3%, 3%, and 2%, respectively, each year. A 1 µg/m^3^ reduction in NO_2_ was, furthermore, estimated to result in a 1% decrease in mortality, myocardial infarction cases, stroke incidence, COPD, and cases of childhood asthma as well as a 0.4% decrease in lung cancer incidence annually.

The potential health impacts of Scenario 2 (a reduction from an average exposure corresponding to Sweden’s Clean Air objective to an average exposure corresponding to the WHO’s 2021 air quality guidelines) for PM_2.5_ included an estimated 4% decrease in mortality, a 12% decrease in myocardial infarction incidence, a 17% decrease in stroke, an 8% decrease in COPD cases, and a 5% decrease in both the incidence of lung cancer and type 2 diabetes annually. Moreover, cases of childhood asthma were estimated to decrease by 14%, the number of women suffering from HDP by 13%, and preterm birth by 10% each year. Considering NO_2_, the findings demonstrated an estimated 7% decrease in mortality, 3% decrease in myocardial infarction cases, 7% decrease in both stroke and COPD incidence, 4% decrease in lung cancer, and 11% decrease in cases of childhood asthma annually.

## Discussion

### Main findings

The results of this HIA using two hypothetical air pollution reduction scenarios, a 1 µg/m^3^ reduction in PM_2.5_ and NO_2_ concentrations, and a reduction from average exposure according to Sweden’s Clean Air objective to the WHO’s 2021 air quality guidelines (a 15 and 20 µg/m^3^ reduction in PM_2.5_ and NO_2_, respectively), illustrated the potential to achieve clear health gains with even small decreases in exposure. Additionally, the findings demonstrated that Sweden’s current Clean Air objective falls short of adequately safeguarding public health.

### Comparison with other HIAs of air pollution

A similar project, “Analytical methods and cost-benefit analysis for the transport sector” (ASEK) has been carried out in Sweden by the Swedish Transport Administration [26]. The ASEK review concluded that mortality, myocardial infarction (incidence), stroke (incidence), COPD (incidence), type 2 diabetes (incidence), childhood asthma (incidence), preterm birth, and sick days were relevant outcomes to include in their evaluation of PM_2.5_’s health effects [26]. The main differences between the present study and ASEK are that the present study included additional health outcomes related to NO_2_ exposure and HDP, and it also excluded sick days. The health economic assessment of Public Health England, upon which ASEK was based, also differs somewhat from the present study: the British assessment includes dementia [27], whereas the present study considered HDP instead. The associations between both of these health outcomes and air pollution exposure are still surrounded by a degree of uncertainty. Additionally, authors chose to include low birth weight instead of preterm birth, as was done in the present study. The weight of evidence for these birth outcomes is similar; to avoid double-counting, however, they should not be included simultaneously, given their close relation to each other. Public Health England further included type 2 diabetes in relation to NO_2_ exposure [27], but this outcome was only considered for PM_2.5_ in the present study. Overall, the ERFs utilized in the present study varied somewhat compared with those applied in ASEK and the British health economic assessment. Moreover, an updated version of the U.S. EPA’s ISA for PM that designates greater evidence for the long-term effects of PM_2.5_ on lung cancer (from “suggestive of, but not sufficient to infer, a causal relationship” to “likely to be a causal relationship”) and effects on the nervous system overall (“likely to be a causal relationship”), with the strongest evidence on cognitive effects among older adults, has been published [28]. The inclusion of lung cancer in the present study is, therefore, further supported. This updated ISA also supports the estimation of the effects of exposure to PM_2.5_ on dementia despite external uncertainties. With this, the health outcomes appropriate to include in HIAs or health economic assessments are not static but, rather, change and expand over time because the state of evidence and scientific knowledge of these associations are continuously developing and improving.

### Methodological considerations

The present study has several strengths. To begin, the demographic (age and sex) distribution of the study population as well as baseline health data were obtained from high-quality databases maintained by governmental agencies (Statistics Sweden and the Swedish National Board of Health and Welfare, respectively). When such data were not available or complete, they were supplemented with data from relevant Sweden-based studies. Additionally, ERFs were based on the most recent and relevant systematic reviews and meta-analyses available in the current literature where possible. Prioritization was also given to studies involving low-exposure settings to identify ERFs more applicable to the state of air pollution in Sweden.

Limitations are also present. For instance, uncertainty in the calculations exists, particularly regarding the ERFs. For instance, even if systematic reviews and meta-analyses were prioritized, discrepancies in ERFs between studies can still arise. Growing evidence suggests that the level of detail used in modeling air pollution can influence the magnitude of an ERF. ERFs for PM_2.5_ and mortality, for example, have been demonstrated to vary substantially depending upon a given dispersion model’s spatial resolution [24]. Models with the highest resolution led to a 25% increased risk of death for each 10 µg/m^3^ increase in PM_2.5_ [24]. In the present study, the ERF for PM_2.5_ and mortality derived by Turner et al. (1.26 per 10 µg/m^3^) [12] was utilized, and similar ERFs have been calculated in several other, more recent studies [29], lending weight to its reliability. Conversely, the ERF from Chen and Hoek [25], which has been utilized by other studies as well as by the WHO in their recent update of air quaility guidelines [3], is substantially lower (1.08 per 10 µg/m^3^). Given this variance, both ERFs were applied in the present study when calcualting mortality attributable to PM_2.5_ for Scenario 1. The higher ERF from Turner et al. was not utilized for Scenario 2, however, as it was likely to predominantly reflect the effects of local PM_2.5_ sources, thus its use may have been misleading. Importantly, linear ERFs have been assumed, which may be a simplification. When tested and observed, the lower part of the ERF has sometimes been observed to be steeper [30, 31]. For instance, in Strak et al., the pooled association between PM_2.5_ and mortality is significantly lower if all participants are included (RR = 1.13 for a 5 µg/m3 increase in PM_2.5_), while the corresponding RR for those exposed to <12 ug/m3 of PM_2.5_was 1.30 [30]. Taking into account the supra-linear form of the ERF, as observed in several studies, there are significant differences in the estimated number of deaths attributed to PM_2.5_. If, for example, near-source PM_2.5_ did have stronger adverse health effects than regional PM_2.5_ [24], any HIAs of air pollution reductions would have to consider whether the decrease in exposure occurred at the local level or in long-range, in-transported particles. This was outside the scope of the present study but is illustrated by the different ERFs’ varying results.

Also important to note is that the choice of baseline mortality will influence the results. This metric differs over time and space and can even differ within a city, as low socio-economic areas typically have lower life expectancies and higher baseline mortality. The choice of baseline mortality in the present study constituted an average of the Swedish population, which means that the estimated health gains from cleaner air might be underestimated for populations of low socio-economic status and over-estimated for populations of high socio-economic status. Additionally, while accurately reflective of conditions in some urban settings, Scenario 2 is not intended to showcase the potential health gains for the entire Swedish population. On the contrary, Swedes are, on average, exposed to air pollution levels below the Clean Air objective, but urban populations often exceed this threshold. Scenario 2 can also be applied to similar demographic settings outside of Sweden, where average exposure to PM_2.5_ and NO_2_ lies at around 10 and 20 µg/m^3^, respectively, and is reduced to adhere to the WHO air quality guidelines, to illustrate potential health improvements. Therefore, the calculations in Scenario 2 should be interpreted in the context of these specific populations. Finally, health impact analyses typically estimate the number of additional cases of a particular health outcome that would occur given a higher (or increased) exposure concentration compared with a lower one, which can also be calculated as a percent increase due to the exposure. In the present study, a percent decrease from the higher value was, instead, quantified, which generates a slightly lower relative value. The resulting estimates are, therefore, more conservative than if the relative differences were calculated using traditional methods.

## Implications

The findings demonstrated significant health benefits associated with enhancing air quality in Sweden, even with a modest decrease of just 1 µg/m^3^ in annual mean PM_2.5_ levels. For municipalities, which are responsible for air quality at the local level in Sweden, the findings underscore the significant health gains achievable through targeted reductions in local sources of air pollution. It is important to recognize, however, that the selected health outcomes included in the present study should not be viewed as representing the entire health burden associated with air pollution. The range of health outcomes influenced by air pollution continues to expand as research advances. It is highly probable that additional health outcomes not investigated in the present study, such as dementia, which is increasingly being considered in HIAs of air pollution exposure, will also be affected by reductions in air pollution [27].

## Conclusion

The current health impact analysis highlights significant health benefits from achieving even minor enhancements in air quality in Sweden.

## Supplemental Material

sj-docx-1-sjp-10.1177_14034948241264099 – Supplemental material for Projected health benefits of air pollution reductions in a Swedish population Supplemental material, sj-docx-1-sjp-10.1177_14034948241264099 for Projected health benefits of air pollution reductions in a Swedish population by Anna Oudin, Erin Flanagan and Bertil Forsberg in Scandinavian Journal of Public Health
