# Supplementary material for: Projected health benefits of air pollution reductions in a Swedish population
Source: Scand J Public Health. 2024 Nov 26;54(1):56–63. doi: 10.1177/14034948241264099 (PMC12858656; doi:10.1177/14034948241264099)
Supplement: sj-docx-1-sjp-10.1177_14034948241264099 – Supplemental material for Projected health benefits of air pollution reductions in a Swedish population [file sj-docx-1-sjp-10.1177_14034948241264099.docx]

Supplementary materials

**Supplementary table S1.** Age distribution of Swedish population (N) by sex for year 2019; data derived from Statistics Sweden. From this, the number of persons in different age groups and risk groups could be calculated (2–18 years old, 15+ years old, 30+ years old, 35+ years old, and 50+ years old)

|  | **Total** |
| --- | --- |
| 0–4 years of age |  |
| Men | 309,567 |
| Women | 292,151 |
| 5–9 years of age |  |
| Men | 320,985 |
| Women | 303,099 |
| 10–14 years of age |  |
| Men | 313,352 |
| Women | 295,667 |
| 15–19 years of age |  |
| Men | 296,072 |
| Women | 272,837 |
| 20–24 years of age |  |
| Men | 310,624 |
| Women | 277,075 |
| 25–29 years of age |  |
| Men | 379,985 |
| Women | 359,711 |
| 30–34 years of age |  |
| Men | 367,181 |
| Women | 348,710 |
| 35–39 years of age |  |
| Men | 333,895 |
| Women | 316,703 |
| 40–44 years of age |  |
| Men | 321,942 |
| Women | 309,321 |
| 45–49 years of age |  |
| Men | 338,208 |
| Women | 328,884 |
| 50–54 years of age |  |
| Men | 344,164 |
| Women | 333,591 |
| 55–59 years of age |  |
| Men | 313,351 |
| Women | 306,757 |
| 60–64 years of age |  |
| Men | 284,848 |
| Women | 283,542 |
| 65–69 years of age |  |
| Men | 267,106 |
| Women | 272,942 |
| 70–74 years of age |  |
| Men | 273,394 |
| Women | 287,021 |
| 75–79 years of age |  |
| Men | 205,253 |
| Women | 223,345 |
| 80–84 years of age |  |
| Men | 121,578 |
| Women | 151,472 |
| 85–89 years of age |  |
| Men | 63,989 |
| Women | 99,640 |
| 90–94 years of age |  |
| Men | 24,740 |
| Women | 51,904 |
| 95–99 years of age |  |
| Men | 5,205 |
| Women | 15,571 |
| 100+ years of age |  |
| Men | 375 |
| Women | 1,832 |
